# Development of an Electrogenerated Chemiluminescence Biosensor using Carboxylic acid-functionalized MWCNT and Au Nanoparticles

**DOI:** 10.3390/s90301662

**Published:** 2009-03-10

**Authors:** Ming-Hua Piao, Dae-Soo Yang, Kuk-Ro Yoon, Seung-Ho Lee, Seong-Ho Choi

**Affiliations:** Department of Chemistry, BK 21 NanoBiosensor Research Team, Hannam University, Daejeon 305-811, Republic of Korea; E-mail: mhpiao@hnu.kr (M. P); ydsfff@hanmail.net (D. Y); kryoon@hnu.kr (K. Y); slee@hnu.kr (S. L)

**Keywords:** Ru(bpy)_3_^2+^, Electrogenerated chemiluminescence, COOH-functionalized MWCNT, Au nanoparticle, ethanol, biosensor, real sample

## Abstract

A COOH-F-MWCNT-Nafion-Ru(bpy)_3_^2+^-Au-ADH electrogenerated chemiluminescence (ECL) electrode using COOH-functionalized MWCNT (COOH-F-MWCNT) and Au nanoparticles synthesized by the radiation method was fabricated for ethanol sensing. A higher sensing efficiency for ethanol for the ECL biosensor prepared by PAAc-*g*-MWCNT was measured compared to that of the ECL biosensor prepared by PMAc-*g*-MWCNT, and purified MWCNT. Experimental parameters affecting ethanol detection were also examined in terms of pH and the content of PAAc-*g*-MWCNT in Nafion. Little interference of other compounds was observed for the assay of ethanol. Results suggest this ECL biosensor could be applied for ethanol detection in real samples.

## 1. Introduction

The detection and quantification of alcohol with high sensitivity, selectivity, and accuracy are required in many different fields from the food industry and clinic analysis, to investigation of adulterations and monitoring of natural processes [1]. Many analytical methods have been developed in recent years for the determination of ethanol such as titration [2], colorimetric methods [3], spectrometric and chromatographic methods [4,5]. Though some of these methods are accurate, they are quite complex, time consuming and often require expensive instrumentation. The nature and specificity of enzyme catalytic activity makes it an excellent tool for chemical analysis. Therefore, an enzyme biosensor could be an excellent alternative for the detection of alcohol.

Owing to the unique properties of nanomaterials, direct electrochemistry and catalytic activity of many enzymes have been observed at electrodes modified with various nanomaterials such as metal oxide nanoparticles, metal nanoparticles, carbon nanotubes, and others [6–10]. The sensitivity and performance of biosensors are being improved by using nanomaterials for their construction. Various nanostructures have been examined as hosts for enzyme immobilization via approaches including protein adsorption, covalent attachment, enzyme encapsulation, and sophisticated combinations of methods. Nanomaterials can not only provide a friendly surface for the assembly of enzyme molecules, but also enhance the electrical-transfer process between enzyme molecules and electrode.

Many enzymes have been employed to prepare various kinds of biosensors using carbon nanotubes (CNTs) [11–15]. Usually, enzymes are immobilized onto CNTs by physical adsorption [16] and covalent bonding [17,18]. In order to immobilize enzymes onto CNTs, CNTs must have hydrophilic properties, and not remain in its hydrophobic state, to results in interaction between enzymes and the surface of CNTs. Radiation-induced graft polymerization (RIGP) is a beneficial method for introduction of functional groups into different polymer materials using specially selected monomers. There have been several reports about RIGP of polar monomers onto polymer substrates to obtain hydrophilic properties for various applications [19–21]. The RIGP method can easily functionalize the surface of CNTs as desired. However, little has been reported about the functionalization of CNTs by RIGP.

Nanometer-sized colloidal gold particles have been reported to adsorb redox enzymes and proteins without any loss of their biological activity [22–25]. In addition, colloidal gold nanoparticles are widely used as a model system because of their ease of synthesis and surface modification [23], good biocompatibility [26], as well as their ability to act as tiny conduction centers which facilitate electron transfer [27]. On the other hand, Au atoms are produced in solution by radiation-induced reduction of Au ion-precursors without chemical reducing agents [28,29]. The species arising from the radiolysis of water, solvated electrons, e_aq_^−^, and H· atoms are the strongest reducing agents. They easily reduce Au ions, producing Au nanoparticles.

The cost-effective regenerable Ru(bpy)_3_^2+^-based ECL sensor has grown in importance since it allows the detection of analytes at low concentrations over a wide linear range and gives an extremely low background signal [30]. Immobilization of Ru(bpy)_3_^2+^ onto a solid electrode surface could not only save on expensive reagents, but also simplify the experimental design. Therefore, many efforts have been developed for the immobilization of Ru(bpy)_3_^2+^ on a variety of composite films [31]. A composite CNT-Nafion film was used for a Ru(bpy)_3_^2+^ ECL sensor [32]. A CNT-titania-Nafion composite film showed improved sensitivity and long term stability compared to the ECL sensor without CNT, and the pure Nafion film [33]. However, a new matrix for the fabrication of the highly sensitive and stable Ru(bpy)_3_^2+^ ECL biosensor is still needed.

In this study, we functionalized MWCNTs by radiation-induced graft polymerization (RIGP) of vinyl monomers with carboxylic acid groups in order to immobilize Ru(bpy)_3_^2+^ and improve solubility and sensitivity. We also prepared the Au nanoparticles by radiation-induced reduction of Au ions in aqueous solution for immobilization of enzymes. An ECL biosensor was fabricated using COOH-F-MWNT and Au nanoparticles as follows: (1) the ECL biosensor was fabricated by immobilization of Au nanoparticles after the immobilization of the Ru(bpy)_3_^2+^ on the surface of COOH-F-MWCNT-Nafion composite film, which was prepared by hand-casting of COOH-F-MWCNT-Nafion composite on the surface of the GC electrode. (2) ADH was adsorbed on COOH-F-MWCNT-Nafion-Ru(bpy)_3_^2+^-Au composite film in phosphate buffer (pH=7.5) for 12 hr at 4.0 °C in order to use it for ethanol sensing. The newly prepared ECL biosensor was characterized by scanning electron microscopy (SEM), and water contact analysis. Furthermore, the sensing efficiency and stability of ethanol to the prepared ECL biosensor were examined. Interference effects of additive compounds for the assay of ethanol were observed on the ECL biosensor. The prepared ECL biosensor was applied for ethanol detection in Soju (a Korean distilled alcoholic beverage) and beer.

## 2. Experimental

### 2.1. Reagents

Acrylic acid (AAc), methacrylic acid (MAc), Nafion (perfluoinated ion-exchange resin, 5% w/v solution in a solution of 90% aliphatic alcohol/10% water mixture), tris(2,2’bipyridyl)-dichlororuthenium (II) (Ru(bpy)_3_^2+^, 98%), alcohol dehydrogenase (ADH), and nicotinamide adenine dinucleotide (NAD^+^) were purchased from Aldrich. MWCNT (95% pure, 10 nm in diameter, and 10 um in length) were obtained from Hanwha Nanotech Co., Ltd (Korea). Solutions for the experiments were prepared with water purified in a Milli-Q puls water purification system (Millipore Co. Ltd., the final resistance of water was 18.2 MΩcm^−1^) and degassed prior to each measurement.

### 2.2. Preparation of the COOH-F-MWCNT by RIGP

MWNTs were purified to remove the catalyst and non-crystallized carbon impurities. MWNTs were treated with a H_2_SO_4_/HNO_3_=3/1 (vol-%) mixture, and in the process MWNTs were cut into shorter segments [34]. The purified and cut MWNTs were used as the supporting materials for grafting of AAc and MAc, respectively. The MWNTs (2.0g) and AAc (2.0g) were mixed in aqueous solution (20 mL). Nitrogen gas was bubbled through the solution for 30 min to remove any residual oxygen gas, and the solution was irradiated by γ-rays from a Co-60 source under atmospheric pressure and ambient temperature. A total irradiation dose of 30 kGy (dose rate =1.0 × 10^4^ Gy/h) was used. The poly(MAc)-grafted MWNTs were prepared using a similar procedure.

### 2.3 Preparation of Au Nanoparticles by the Radiation-induced Reduction Method

Hydrogen tetrachloroaurate(III) was dissolved in water with 2-propanol and PVP to form a primary solution with a predetermined concentration of the metal salts (HAuCl_4_ = 2.0 ×10^−3^ M). Nitrogen gas was bubbled through the solution for 30 min to remove oxygen gas, and the solution was irradiated with γ-rays from the Co-60 source under atmospheric pressure and ambient temperature. A total irradiation dose of 30 kGy (dose rate =1.0 × 10^4^ Gy/h) was used. The reddish gold colloids were prepared as a homogenous solution and the size the gold nanoparticles was about 100 nm.

### 2.4. Fabrication of ECL Biosensor

**Figure 1.**
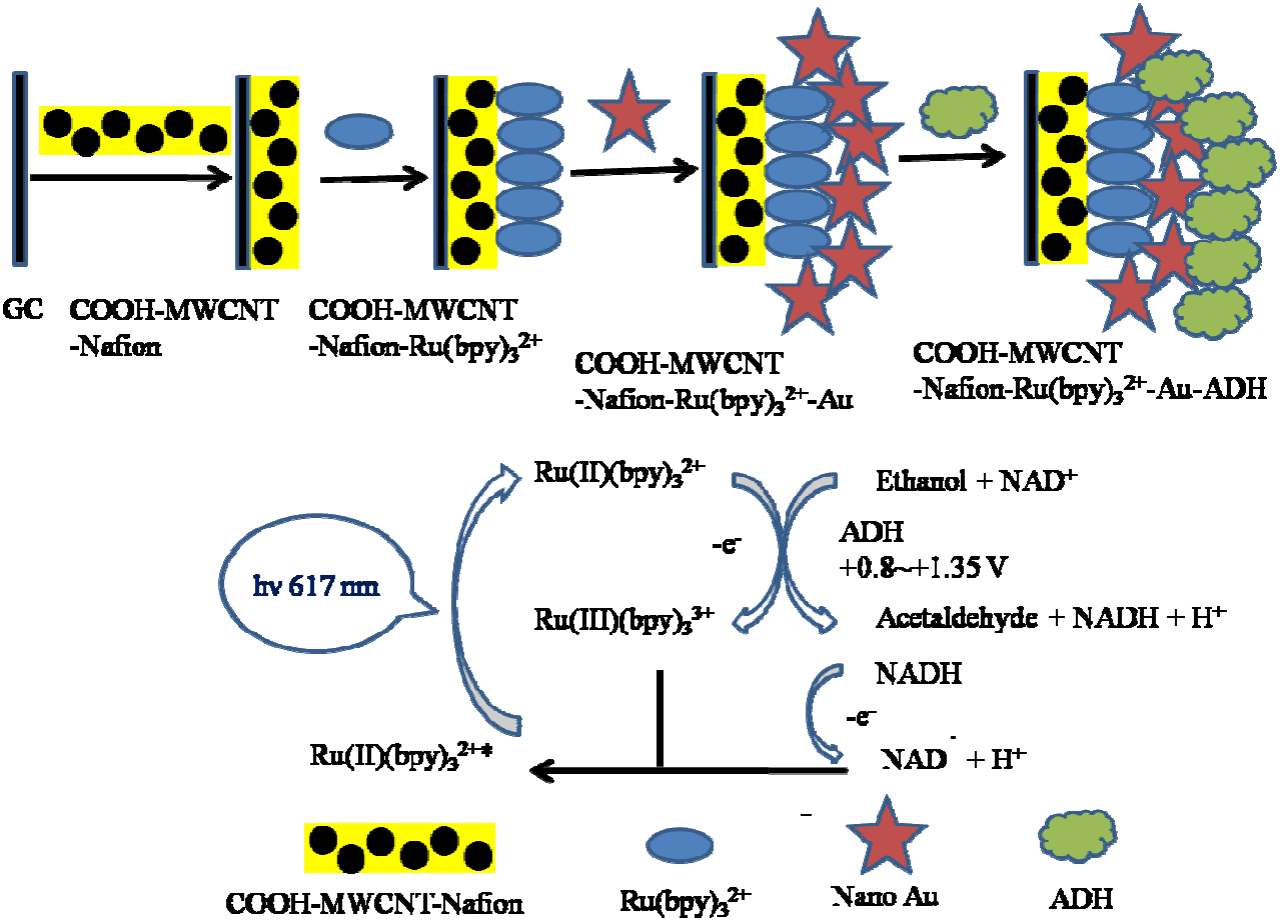
Schematic illustration of the ECL biosensor fabrication procedure.

Figure 1 shows the preparation procedure of the ECL alcohol sensor. The COOH-MWCNT-Nafion composite solution (0.5 mg·mL^−1^) was prepared by dispersion of COOH-MWCNT in Nafion. An aliquot (3 μL) from the above solution was hand-cast on the surface of a pre-cleaned glassy carbon electrode. The thin composite film was dried for 3 hr at room temperature (thickness: 200 nm). Subsequently, the COOH-MWCNT-Nafion electrode was then immersed in a 2 mM Ru(bpy)_3_^2+^ solution in 0.1 M phosphate buffer solution (pH 7.0) for 5 hr. The COOH-MWCNT-Nafion-Ru(bpy)_3_^2+^ electrode was immersed in 10 mM Au nanoparticle solution which was γ-irradiated for 4 hr. Finally, the COOH-MWCNT-Nafion-Ru(bpy)_3_^2+^-Au electrode was immersed in 1 mg·mL^−1^ alcohol dehydrogenase solution for 12 hr at 4 °C. The prepared electrode was then kept in the refrigerator until use.

### 2.5. Instrumentation

Cyclic voltammetric experiments were performed with a model 283 Potentiostat/Gavanostat (Ametek PAR, U.S.A). All experiments were carried out with a conventional three-electrode system. The working electrode was glassy carbon (diameter: 0.2 mm) coated with the composite films. The counter electrode was Pt wire, and the reference electrode was Ag/AgCl (sat’d KCl). The ECL experiment was equipped with a photomultiplier tube (PMT) system [35]. The surfaces of COOH-MWCNT-Nafion composite films and Au nanoparticles were studied by transmission electron microscopy (TEM) (Tecnai G2 Spirit, FEI, USA). Alcoholdehydrogenase absorption was confirmed by scanning electron microscopy (SEM, FE-SEM, JSM-7000F, JEOL Ltd., Japan). Contact angle measurements were performed using a Phoenix 300 goniometer (Surface Electro Optics Co., Ltd., Korea).

## 3. Results and Discussion

**Figure 2.**
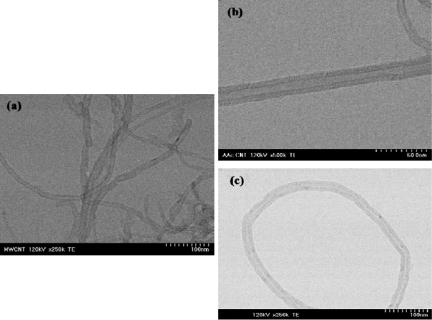
TEM images of the purified MWCNT (a), PAAc-g-MWCNT (b), and PMAc-g-MWCNT (c) prepared by γ-irradiation.

Radiation-induced graft polymerization (RIGP) is a useful method for introduction of functional groups into different polymer materials using specially selected monomers. There have been several reports about RIGP of polar monomers onto polymer subtracts to obtain hydrophilic properties for versatile applications [19–21]. The RIGP method can easily functionalize the surface of MWNTs. We performed the RIGP of AAc and MAc on the purified MWCNTs in aqueous solution. We selected AAc and MAc because of the hydrophobic properties of the vinyl group and hydrophilic properties of COOH group on these monomers. The AAc and MAc monomer vinyl groups face the surface of MWCNTs because of their hydrophobic properties, while the COOH groups of the monomers face the aqueous solution because of their hydrophilic properties. The radical polymerization of AAc and MAc monomers occurred on the surface of MWNTs during γ-irradiation. As a result, the COOH group was immobilized on the surface of MWNTs as tubular-type as shown in Figure 2.

In the TEM image of Figure 2, the diameters of the purified MWCNT, AAc-*g*-MWCNT and the coated MWNTs were at 11 nm, 20 nm, and 21 nm, respectively. According to these results, we successfully prepared the COOH-MWNTs for use as a biosensor.

**Figure 3.**
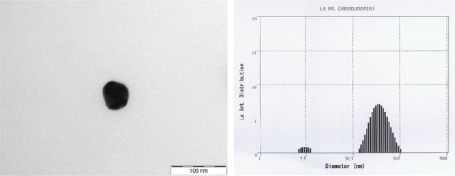
EF-TEM image (left) and ELS spectrum (right) of PVP-protected Au nanoparticle prepared by radiation-induced reduction method.

Figure 3 exhibits the TEM image and ELS spectrum of the PVP-stabilized Au nanoparticles prepared by radiation-induced reduction method. TEM image shows that a uniform size of Au nanoparticles of ∼50 nm was achieved by γ-radiation-induced reduction of Au nanoparticles. ELS spectra exposed two size distribution regions. This distribution is considered a result of primary Au particles and secondary Au particles which are aggregated from primary Au particles. We prepared the biosensor for detection of alcohol using COOH-MWCNTs and Au nanoparticle prepared by γ-irradiation.

**Figure 4.**
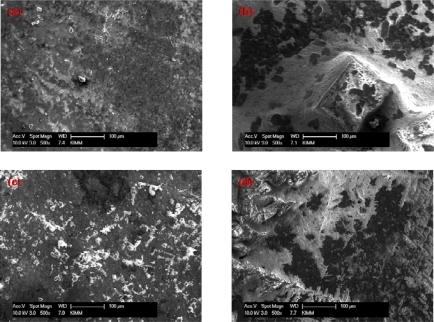
SEM images of composite film on glassy carbon. (a) PAAc-g-MWCNT-Nafion, (b) PAAc-g-MWCNT-Nafion-Ru(bpy)_3_^2+^, (c) PAAc-g-MWCNT-Nafion-Ru(bpy) _3_^2+^-Au, and (d) PAAc-g-MWCNT-Nafion-Ru(bpy) _3_^2+^-Au-ADH.

Figure 4 shows the SEM images of the composite film on glassy carbon. (a) PAAc-*g*-MWCNT-Nafion, (b) PAAc-*g*-MWCNT-Nafion-Ru(bpy)_3_^2+^, (c) PAAc-*g*-MWCNT-Nafion-Ru(bpy)_3_^2+^-Au, and (d) PAAc-*g*-MWCNT-Nafion-Ru(bpy)_3_^2+^-Au-ADH. Upon loading ADH, a few smaller features on the surface with a different roughness appear in this electrode in comparison to the pre-ADH-loaded electrode surface. From these results, we conclude that Ru(bpy)_3_^2+^, Au, and ADH were well immobilized on the surface of the GC electrode.

**Figure 5.**
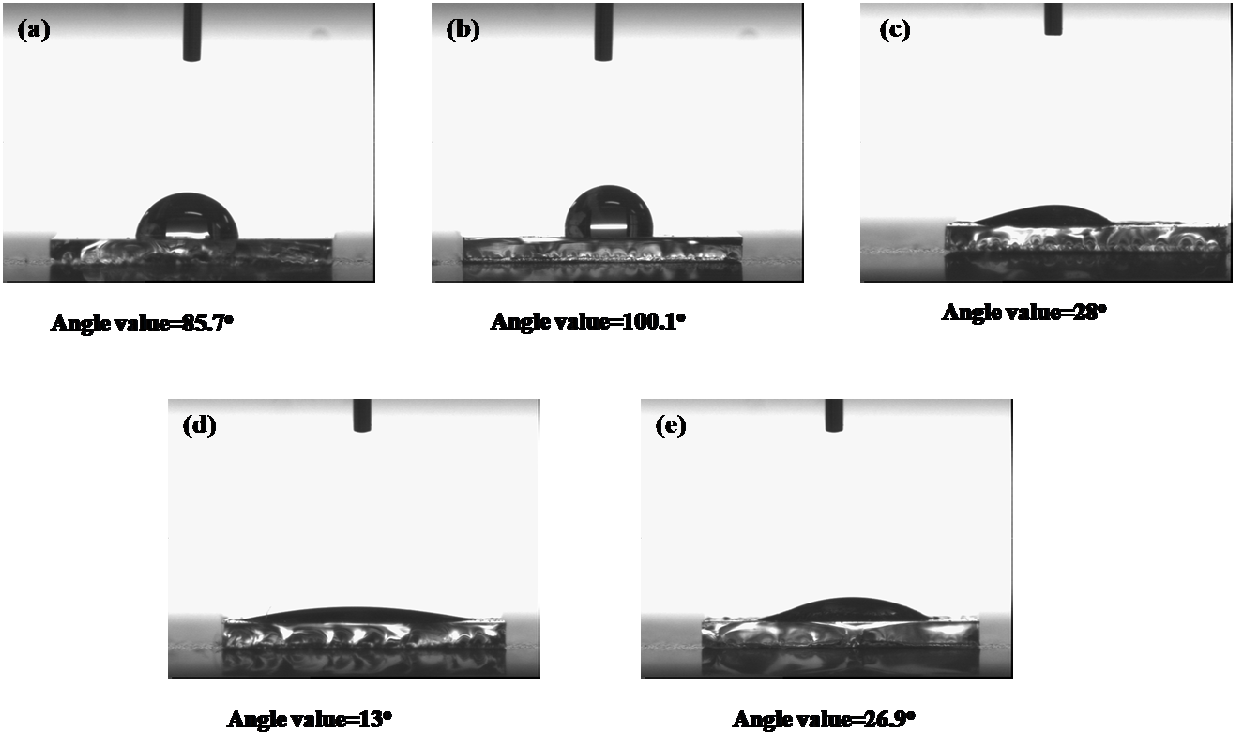
Water contact angle for the composite film on ITO glass. (a) bare, (b) PAAc-g-MWCNT-Nafion, (c) PAAc-g-MWCNT-Nafion-Ru(bpy) _3_^2+^, (d) PAA-g-MWCNT-Nafion-Ru(bpy) _3_^2+^-Au, and (e) PMAc-g-MWCNT-Nafion-Ru(bpy) _3_^2+^-Au-ADH.

In order to confirm immobilization of ADH on the surface of the composite film, we measured the water contact angle, as shown in Figure 5. This figure represents the water contact images for the composition films: (a) bare, (b) PAAc-*g*-MWCNT-Nafion, (c) PAAc-*g*-MWCNT-Nafion-Ru(bpy)_3_^2+^, (d) PAAc-*g*-MWCNT-Nafion-Ru(bpy)_3_^2+^-Au, (e) PAAc-*g*-MWCNT-Nafion-Ru(bpy)_3_^2+^-Au-ADH. We used ITO glass as model subtracts mimicking the GC electrode. The contact angle of water at the ITO glass surface was 75°. An increase in contact angle (100.1°) was noticed with a deposition of PAAc-*g*-MWCNT-Nafion. This indicates that the surface properties of the ITO glass after coating PAAc-*g*-MWCNT-Nafion changed to hydrophobic. Decreases in contact angle were noticed after immobilizing Ru(bpy)_3_^2+^, Au, and ADH on the PAAc-*g*-MWCNT-Nafion composite film. This indicates that the surface of the ITO glass after immobilizing Ru(bpy)_3_^2+^, Au, and ADH on the PAAc-*g*-MWCNT-Nafion composite film made it hydrophilic. The resulting water contact angles show that the ADH-immobilized biosensor was successfully fabricated using COOH-MWCNTs and Au nanoparticles prepared by the γ-irradiation technique.

**Figure 6.**
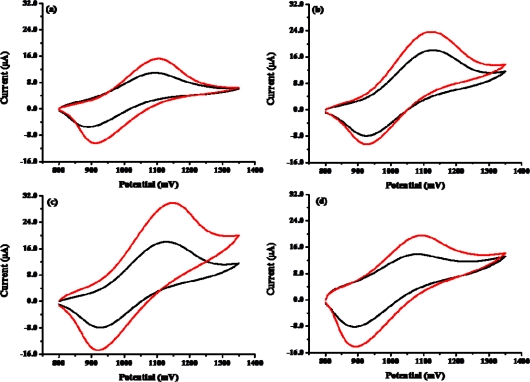
Cyclic voltammograms of biosensors in the absence (black) and presence (red) of 1×10^−3^ M ethanol in 50mM phosphare buffer (pH 7.5) with a scan rate of 100 mV/s. (a) Nafion-Ru(bpy) _3_^2+^-Au-ADH, (b) purified MWCNT-Nafion-Ru(bpy) _3_^2+^-Au-ADH, (c) PAAc-*g*-MWCNT-Nafion-Ru(bpy) _3_^2+^-Au-ADH, and (d) PMAc-*g*-MWCNT-Nafion-Ru(bpy) _3_^2+^-Au-ADH.

**Figure 7.**
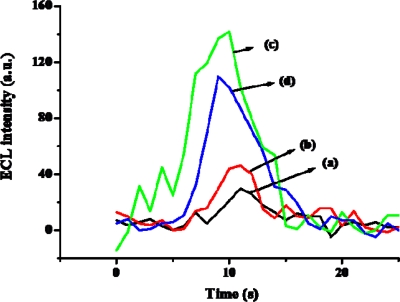
ECL intensity of the prepared biosensor using various nanomaterials in phosphate buffer (pH 7.5) containing 1.0×10^−3^ M ethanol with the scan rate of 100 mV/s. (a) Nafion-Ru(bpy)_3_^2+^-Au-ADH, (b) purified MWCNT-Nafion-Ru(bpy)_3_^2+^-Au-ADH, (c) PAAc-*g*-MWCNT-Nafion-Ru(bpy)_3_^2+^-Au-ADH, and (d) PMAc-*g*-MWCNT-Nafion-Ru(bpy)_3_^2+^-Au-ADH.

In order to determine biosensor sensitivity due to nanomaterials, we fabricated the biosensor with COOH-MWCNTs and without COOH-MWCNTs. Figures 6 and 7 show the cyclic voltammograms (CVs) and ECL spectra of the biosensor in the absence and presence of 1×10^−3^ M ethanol and 8×10^−4^ M NAD^+^ in PBS (pH 7.5) with the scan rate of 100 mV/s: (a) Nafion-Ru(bpy)_3_^2+^-Au-ADH, (b) the purified MWCNT-Nafion-Ru(bpy)_3_^2+^-Au-ADH, (c) PAAc-g-MWCNT-Nafion-Ru(bpy)_3_^2+^-Au-ADH, and (d) PMAc-g-MWCNT-Nafion-Ru(bpy)_3_^2+^-Au-ADH. The presence of ethanol resulted in the enzymatic reaction and the generation of NADH, which in turn reacted with Ru(bpy)_3_^3+^ generated on the working electrode. This process finally led to an increase of oxidation current. As shown in Figure 6, a higher oxidation current was observed at the PAAc-*g*-MWCNT-Nafion-Ru(bpy)_3_^2+^-Au-ADH electrode compared with the Nafion-Ru(bpy)_3_^2+^-Au-ADH, purified MWCNT-Nafion-Nafion-Ru(bpy)_3_^2+^-Au-ADH, and PMAc-*g*-MWCNT-Nafion Nafion-Ru(bpy)_3_^2+^-Au-ADH electrodes. So, we used the PAAc-*g*-MWCNT-Nafion-Ru(bpy)_3_^2+^-Au-ADH electrode in a further experiment. Basically, alcohol dehydrogenase required the addition of NAD^+^ as a cofactor for enzymatic reaction so that NAD^+^ was reduced to NADH simultaneously with substrate oxidation. Then NADH could lose one electron and one proton to generate NAD. radical on the electrode. The produced NAD. radical subsequently was reacted with Ru(bpy)_3_^3+^, which was produced *in situ* on the working electrode to generate excited state Ru(bpy)_3_^2+^* which went back to ground state, giving off light, and at the same time Ru(bpy)_3_^2+^ was reproduced as described in Figure 1. As shown in Figure 7, a higher ECL intensity was observed at the PAAc-*g*-MWCNT-Nafion-Ru(bpy)_3_^2+^-Au-ADH electrode compared with the Nafion-Ru(bpy)_3_^2+^-Au-ADH, purified MWCNT-Nafion-Nafion-Ru(bpy)_3_^2+^-Au-ADH, and PMAc-*g*-MWCNT-Nafion Nafion-Ru(bpy)_3_^2+^-Au-ADH electrode.

**Figure 8.**
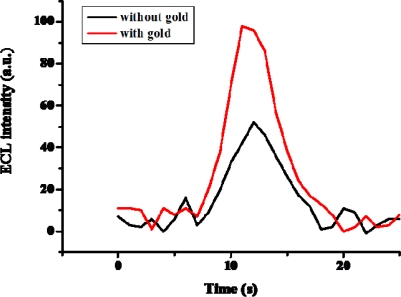
ECL intensity of PAAc-*g*-MWCNT-Nafion-Ru(bpy)_3_^2+^-ADH electrode in 50 mM phosphate buffer containing 1×10^−3^ M ethanol (pH 7.5) with a scan rate of 100 mV/s. (black) without, and (red) with gold immobilization.

The ECL intensity before and after Au nanoparticle immobilization was compared. From Figure 8, we observe that the Au nanoparticle-immobilized electrode showed two times higher ECL intensity than that of the electrode without immobilized Au nanoparticles. Results show that the ADH was well-immobilize on the surface of the GC electrode using Au nanoparticles as linker.

**Figure 9.**
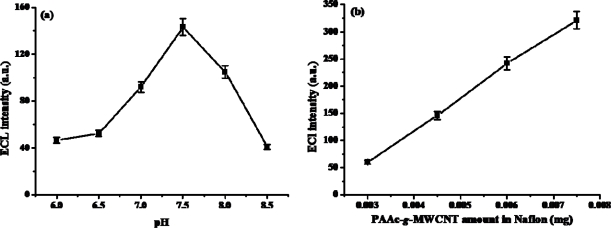
The effect of pH (a) and PAAc-*g*-MWCNT amount in Nafion composite films (b) on the ECL intensity. The ECL intensity was measured in phosphate buffer solution containing 1.0×10^−3^ M ethanol. The points shown are the mean of 3 determination (±S.D.)

The effect of pH on the response of the PAAc-*g*-MWCNT-Nafion-Ru(bpy)_3_^2+^-Au-ADH biosensor was examined by comparing the anodic peak current to 1×10^−3^ M ethanol in the range of pH 5.5 ∼ 8.5 with the scan rate of 100 mV/s. Figure 9-a shows the pH profile obtained from this experiment. The anodic peak current increased from pH 5.5 to 7.5, and decreased at higher pH. This may be due to the fact that NAD^+^ was unstable in alkaline solution [36]. Thus, the modified biosensor showed highest efficiency at pH 7.5, so we used this pH condition in all experiments. The effects of PAAc-*g*-MWCNT content in the Nafion on ECL intensity were observed. The COOH-MWCTs level is a very important factor for increasing ECL intensity for a biosensor because the COOH-MWCNTs can be immobilized with Ru ions. So, we examined increasing amounts of PAAc-*g*-MWCNTs. As is shown in Figure 9-b, the ECL intensity is increased with increasing amounts of PAAc-*g*-MWCNTs because the carboxylic acid group of MWCNTs composite reacts with Ru(bpy)_3_^2+^ ions. However, above 4.5×10^−3^ mg of PAAc-*g*-MWCNTs on the biosensor, the ECL intensity rapidly decreases by about 50%. We supposed that the PAAc-*g*-MWCNTs were not well dispersed in the Nafion film, so the coating of Nafion film with PAAc-*g*-MWCNTs was not well prepared. We determined an optimum amount (4.5×10^−3^ mg) of PAAc-*g*-MWCNTs in this experiment, and it was used in this and following experiments.

**Figure 10.**
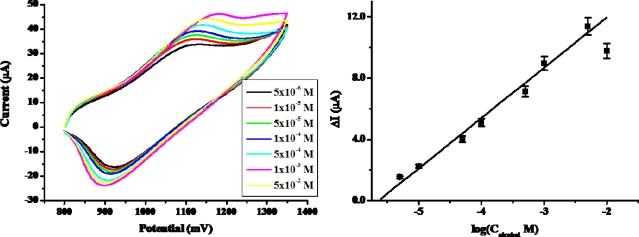
Calibration plot of PAAc-*g*-MWCNTs-Nafion-Ru(bpy)_3_^2+^-Au-ADH electrode for ethanol.

**Figure 11.**
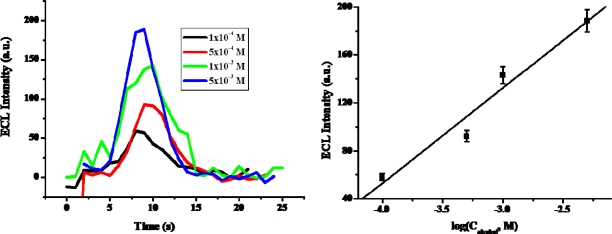
Calibration plot of PAAc-*g*-MWCNT-Nafion-Ru(bpy)_3_^2+^-Au-ADH electrode. Inset: ECL intensity of PAAc-*g*-MWCNT-Nafion-Ru(bpy)_3_^2+^-Au-ADH electrode in different concentration of ethanol.

Figures 10 and 11 show the calibration plots of the prepared PAAc-*g*-MWCNT-Nafion-Ru(bpy)_3_^2+^-Au-ADH electrode for ethanol detection under optimal experimental conditions. The calibration curve was plotted on anodic current via logarithmic alcohol concentration as shown in Figure 10. The anodic current increased with increasing ethanol concentration. The sensitivity of the prepared biosensor for ethanol was 0.3289 μA/M in a range of concentrations from 5.0×10^−6^ M to 5.0×10^−3^ M. The sensitivity of the biosensor prepared with PAAc-*g*-MWCNT was 10-fold higher than that of biosensor prepared by MWCNT and other biosensors [37]. The detection limits were calculated at 1.0×10^−6^ M according to the signal-to-noise (n=3). This is 40 times lower than that of biosensor prepared by MWCNT [38]. On the other hand, the calibration curve was plotted on ECL intensity via logarithmic ethanol concentration as shown in Figure 11. As shown in this figure, the ECL intensity increased with increasing ethanol concentration. The sensitivity of the prepared biosensor in ECL was 7.9438 a.u./M in the linear range from 1.0×10^−4^ to 5.0×10^−3^ M (R^2^=0.979), and the detection limit was 1×10^−5^ M (S/N=3). The linear range and detection limit were comparable with those written of in other papers in which ADH was self-assembling ADH to Ru(bpy)_3_^2+^-AuNPs aggregates on an ITO electrode surface [39].

**Figure 12.**
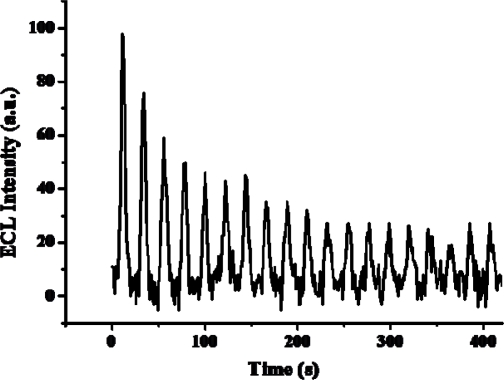
ECL intensity of PAAc-*g*-MWCNT-Nafion-Ru(bpy)_3_^2+^-Au-ADH electrode in phosphate buffer (pH 7.5) containing 5×10^−4^ M ethanol under continuous potential scanning for 19 cycles.

Stability is a very important factor for practical applications, so the stability of the ECL biosensor was investigated under continuously cyclic potential scanning for 19 cycles in PBS (pH=7.5) containing 5.0×10^−4^ M ethanol at the scan rate of 100 mV/s. As shown in Figure 12, the ECL intensity decreased when continuously cycling, observed over the full 19 cycles of continuous cyclic potential scanning.

Interference effects of some common interferents in food and beverages on the assay of ethanol at PAAc-*g*-MWCNT-Nafion-Ru(bpy)_3_^2+^-Au-ADH electrodes were observed. The ratio of ethanol to interferents is 1:1. All interferents tested were present at a concentration of 1 mM. As seen from Table 1, relative responses to ethanol in the presence of citric acid, ascorbic acid, acetic acid, and oxalic acid were below 117%. The interference of citric acid and acetic acid was very small. The interference of ascorbic acid and oxalic acid was a little high, but there is much less ascorbic acid and oxalic acid than ethanol in soju and beer. Thus, the PAAc-*g*-MWCNT-Nafion-Ru(bpy)_3_^2+^-Au-ADH electrode could be applied for the detection of ethanol in real samples. As a simple application of the PAAc-*g*-MWCNT-Nafion-Ru(bpy)_3_^2+^-Au-ADH electrode for the analysis of real samples, the determination of ethanol was carried out in commercial beer and soju. Three-fold diluted beer and soju with the supporting electrolyte solution were needed before the cyclic voltammetric measurements and ECL measurements were performed. The determination of ethanol was performed by applying the standard addition method in order to minimize the slight matrix effect observed. The results obtained were 4.5±0.3 for beer and 25±0.2 for soju. As seen here, the obtained results demonstrate fairly well the usefulness of the PAAc-*g*-MWCNT-Nafion-Ru(bpy)_3_^2+^-Au-ADH for the analysis of ethanol in beer and soju.

## 4. Conclusions

An ECL biosensor was fabricated using COOH-F-MWNT and Au nanoparticles as follows: ECL biosensor was fabricated by immobilization of Au nanoparticles after the immobilization of the Ru(bpy)_3_^2+^ on the surface of the COOH-F-MWCNT-Nafion composite film, which was prepared by hand-casting of the COOH-F-MWCNT-Nafion composite on the surface of the GC electrode. Subsequently, ADH was adsorbed on the COOH-F-MWCNT-Nafion-Ru(bpy)_3_^2+^-Au composite film in phosphate buffer. The sensing efficiency of the prepared biosensor for ethanol was investigated. From the results, the conclusions were as follows:

1. The sensing range of the prepared biosensor for ethanol was in the range of 5.0×10^−6^ M −5.0×10^−3^ M on CV detection.
2. The sensing range of the prepared biosensor for ethanol was in the range of 1.0×10^−4^ M −5.0×10^−3^ M on ECL detection.
3. Relative response of ethanol to interference such as citric acid, ascorbic acid, acetic acid, and oxalic acid were below 117%.
4. The prepared biosensor using COOH-F-MWCNT and Au nanoparticles exhibited a wide linear range, high sensitivity, and good stability.
5. The COOH-F-MWCNT prepared by RIGP and Au nanoparticles prepared by radiation-induced reduction can be used for biosensor materials.
6. The prepared biosensor using COOH-F-MWCNT and Au nanoparticles could be used to measure ethanol-levels in soju and beer.

## Figures and Tables

**Table 1.**
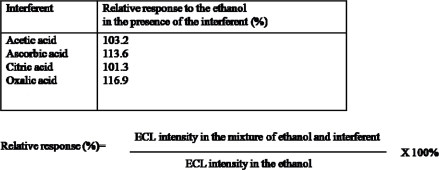
Interference effect of various compounds on the assay of ethanol at PAAc-*g*-MWCNT-Nafion-Ru(bpy)_3_^2+^-Au-ADH electrode. (Ratio of 1:1 ethanol to interferent) (n=3)
